# Regional division and reduction algorithm for minimizing the sum of linear fractional functions

**DOI:** 10.1186/s13660-018-1651-9

**Published:** 2018-03-21

**Authors:** Pei-Ping Shen, Ting Lu

**Affiliations:** College of Mathematics and Information Science, Henan Normal University, Xinxiang, P.R. China

**Keywords:** Global optimization, Sum of linear ratios, Regional division and reduction, Fractional programming

## Abstract

This paper presents a practicable regional division and cut algorithm for minimizing the sum of linear fractional functions over a polyhedron. In the algorithm, by using an equivalent problem (P) of the original problem, the proposed division operation generalizes the usual standard bisection, and the deleting and reduction operations can cut away a large part of the current investigated region in which the global optimal solution of (P) does not exist. The main computation involves solving a sequence of univariate equations with strict monotonicity. The proposed algorithm is convergent to the global minimum through the successive refinement of the solutions of a series of univariate equations. Numerical results are given to show the feasibility and effectiveness of the proposed algorithm.

## Introduction

Consider the following class of fractional programming:

$$ \mbox{(FP)}: \textstyle\begin{cases} \min & \sum_{i=1}^{N}\frac{c^{\top }_{i} y+c_{0i}}{d_{i}^{\top }y+d_{0i}} \\ \mbox{s.t.} & Ay\leq b, y\geq 0, \end{cases} $$

where $A=(a_{rj})_{m \times n}$ is a real matrix, $c_{i}=(c_{ij})_{1 \times n}$ and $d_{i}=(d_{ij})_{1 \times n}$ are real vectors for each $i=1,\ldots ,N$, $b=(b_{r})_{m\times 1}$, $c_{0i},d_{0i} \in R$. In addition, we assume that

$$ \bar{D}= \bigl\{ y\in R^{n} \mid Ay\leq b, y\geq 0 \bigr\} $$

is a nonempty compact set.

Since each $d_{i}^{\top }y+d_{0i}$ is a continuous function on *D̄*, by the intermediate value theorem we can obtain that $d_{i}^{\top }y+d_{0i}>0$ or $d_{i}^{\top }y+d_{0i}<0$ for any $y\in \bar{D}$. Consequently,

$$ \sum_{i=1}^{N}\frac{c^{\top }_{i} y+c_{0i}}{d_{i}^{\top }y+d_{0i}}= \sum _{i\in I^{+}}\frac{c_{i}^{\top }y+c_{0i}}{d_{i}^{\top }y+d_{0i}}+ \sum _{i\in I^{-}}\frac{c_{i}^{\top }y+c_{0i}}{d_{i}^{\top }y+d_{0i}}= \sum_{i\in I^{+}} \frac{c_{i}^{\top }y+c_{0i}}{d_{i}^{\top }y+d_{0i}}+ \sum_{i\in I^{-}}\frac{-(c_{i}^{\top }y+c_{0i})}{-(d_{i}^{\top }y+d _{0i})}, $$

where $I^{+}=\{i \mid d_{i}^{\top }y+d_{0i}>0, i=1,\ldots ,N, \forall y\in \bar{D} \}$ and $I^{-}=\{i \mid d_{i}^{\top }y+d_{0i}<0, i=1, \ldots ,N, \forall y\in \bar{D} \}$. Additionally, notice that

$$\begin{aligned} \min \sum_{i=1}^{N} \frac{c_{i}^{\top }y+c_{0i}}{d_{i}^{\top }y+d_{0i}} & \Longleftrightarrow \min \sum_{i=1}^{N} \biggl( \frac{c_{i}^{\top }y+c_{0i}}{d_{i}^{\top }y+d_{0i}}+\bar{M}_{i} \biggr) \\ & \Longleftrightarrow \min \sum_{i=1}^{N} \frac{c_{i}^{\top }y+c_{0i}+ \bar{M}_{i}(d_{i}^{\top }y+d_{0i})}{d_{i}^{\top }y+d_{0i}} \end{aligned}$$

by choosing a sufficiently large value $\bar{M}_{i}$ ($i=1,\ldots ,N$) with $c_{i}^{\top }y+c_{0i}+\bar{M}_{i}(d_{i}^{\top }y+d_{0i})>0$ for any $y\in \bar{D}$. Therefore, in the following, without loss of generality, we suppose that $c_{i}^{\top }y+c_{0i}>0$ and $d_{i}^{\top }y+d_{0i}>0$, $\forall y\in \bar{D}$, for each $i=1,\ldots ,N$.

Problem (FP) is a well-known class among fractional programming problems. Theoretically, it is NP-hard [1, 2]. The primary challenges in solving problem (FP) arise from a lack of useful properties (convexity or otherwise) and from the number of ratios. In general, problem (FP) possesses more local optimal solutions that are not globally optimal [3], and so problem (FP) owns major theoretical and computational difficulties. From an application point view, this problem has a large deal of applications; for instance, traffic and economic domain [4, 5], multistage stochastic shipping problems [6], data envelopment analysis [7], and queueing-location problems [8]. The reader is referred to a survey to find many other applications [4, 5, 9–11].

Many algorithms have been proposed to solve problem (FP) with a limited number of ratios [4, 12–19]. For instance, Wang and Shen [4] give an efficient branch and bound algorithm by using a transformation technique and the linear relaxation programming of the objective function. By applying Lagrangian duality theory, Benson [9] presents a simplicial branch and bound duality-bounds algorithm. Carlsson and Shi [10] propose a linear relaxation algorithm with lower dimension which is performed on a 2*N*-dimensional domain instead of the original *n*-dimensional one, the computational time is long with larger *N*. Ji and Zhang [16] consider a deterministic global optimization algorithm by utilizing a transformation technique and a linearizing method. Jiao et al. [17, 18] present the branch and bound algorithms for globally solving sum of linear and generalized polynomial ratios problems, by solving a sequence of linear relaxation programming problems. In short, most of them (see [4, 9, 16–18] for example) are branch and bound algorithms. The key idea behind such algorithms mentioned above is that the branch and bound operator is performed on an *N*-dimensional region (or the correspondence relating to *N* and *n*) rather than the native *n*-dimensional feasible set, that is, they all work on a space whose dimension increases with the number *N* of ratios.

In this article, a new division and reduction algorithm is proposed for globally solving problem (FP). To solve problem (FP), an equivalent optimization problem (P), whose objective function is just a simple univariate, is first presented by exploiting the feature of this problem. Then, in order to design a more efficient algorithm, several basic operations: division, deleting, and reduction, are incorporated into a similar branch and bound framework by utilizing the particular structure of problem (P). Compared with the usual branch and bound (BB) methods (e.g., [4, 5, 9, 10, 16]) mentioned above, the goal of this research is three fold. First, the proposed bounding operation is simple since the lower bound of the subproblem of each node can be achieved easily only by arithmetic computations, distinguishing it from the ones obtained by solving convex/linear programs in the usual BB methods. Second, the reduction operation that does not appear in other BB methods is used to tighten the range of each variable, such that the growth of the branch tree can be suppressed. Moreover, the main computational cost of the algorithm is to implement the reduction operation which solves univariate equations with strict monotonicity. Third, the problem in this paper is more general than the others considered in [10, 11], since we only request $d_{i}^{\top }y+d_{0i}\neq 0$ for each *i*. Further, the proposed adaptive division operation both generalizes and is superior to the usual standard bisection in BB methods according to the numerical computational result in Sect. 5. Also, the computational results of the problem with the large number of ratio terms can be obtained to illustrate the feasibility and validity of the proposed algorithm.

This paper is summarized as follows. In Sect. 2, by using a conversion strategies, the original problem (FP) is transformed into an equivalent optimization problem (P). The adaptive division, deleting, and reduction operations are shown in Sect. 3. In Sect. 4 we give the proposed algorithm and its convergence. Some numerical results demonstrate the feasibility and availability of the algorithm in Sect. 5.

## Equivalent problem

For solving problem (FP), we first convert the primary problem (FP) into an equivalent optimization problem (P), in which the objective function is a single variable and the constraint functions are the difference of two increasing functions. To see that such a reformulation is possible, let us denote, for each $i=1,\ldots ,N$,

$$ L_{i}=\frac{1}{\max_{y\in \bar{D}} d_{i}^{\top }y+d_{0i}}, \qquad U_{i}=\frac{1}{\min_{y\in \bar{D}} d_{i}^{\top }y+d_{0i}}. $$

Clearly, $L_{i},U_{i}>0$ for each *i*. Additionally, by introducing some additional variables $w=(w_{1},w_{2},\ldots ,w_{N})\in R^{N}$, from (FP) we then obtain the following equivalent problem:

$$ \mbox{(FP1)}: \textstyle\begin{cases} \min & \sum_{i=1}^{N} w_{i}(c_{i}^{\top }y+c_{0i}) \\ \mbox{s.t.} & 1-w_{i}(d_{i}^{\top }y+d_{0i})\leq 0, \quad i=1,\ldots ,N, \\ &L_{i} \leq w_{i}\leq U_{i}, \quad i=1,\ldots ,N,\\ & y\in \bar{D}. \end{cases} $$

For simplicity, we denote

$$\begin{aligned}& I_{0}^{+}=\{i \mid c_{0i}>0, i=1,\ldots ,N\}, \qquad I_{0}^{-}=\{i \mid c _{0i}< 0, i=1,\ldots ,N \}, \\& J_{i}^{+}=\{j \mid c_{ij}>0, j=1,\ldots ,n\}, \qquad J_{i}^{-}=\{j \mid c _{ij}< 0, j=1,\ldots ,n \}, \\& T_{i}^{+}=\{j \mid d_{ij}>0, j=1,\ldots ,n\}, \qquad T_{i}^{-}=\{j \mid d _{ij}< 0, j=1,\ldots ,n \}, \\& M_{r}^{+}=\{j \mid a_{rj}>0, j=1,\ldots ,n\}, \qquad M_{r}^{-}=\{j \mid a _{rj}< 0, j=1,\ldots ,n \}. \end{aligned}$$

Define a box as follows:

$$ [\underline{y},\bar{y}]= \Bigl\{ y \in R^{n} \mid y_{j}^{l} \triangleq \min_{y\in \bar{D}} y_{j} \leq y_{j} \leq y_{j}^{u}\triangleq \max_{y\in \bar{D}} y_{j}, j=1,\ldots ,n \Bigr\} . $$

Based on the above notations, by introducing an extra variable $y_{0}\in R^{+}$, we can convert the above problem (FP1) into the form:

$$ \mbox{(FP2)}: \textstyle\begin{cases} \min & y_{0} \\ \mbox{s.t.} & \sum_{i=1}^{N} \sum_{j\in J_{i}^{+}} c_{ij}w_{i}y_{j} \\ &\quad {}+\sum_{i\in I_{0}^{+}} c_{0i}w_{i}-\sum_{i=1}^{N} \sum_{j\in J_{i}^{-}} (-c_{ij})w_{i}y_{j}-\sum_{i\in I_{0}^{-}} (-c_{0i})w_{i}-y_{0}\leq 0, \\ &-\sum_{j\in T_{i}^{+}}d_{ij}w_{i}y_{j}+\sum_{j\in T_{i}^{-}}(-d_{ij})w_{i}y_{j}-d_{0i}w_{i}+1 \leq 0, \quad i=1,\ldots ,N, \\ & \sum_{j\in M_{r}^{+}}a_{rj}y_{j}-b_{r}-\sum_{j\in M_{r}^{-}}(-a_{rj})y_{j}\leq 0, \quad r=1,\ldots ,m, \\ & L_{i} \leq w_{i}\leq U_{i},\quad i=1,\ldots ,N, \\ & y\in [\underline{y},\bar{y}], \qquad y_{0}\in [y_{0}^{l},y_{0}^{u}], \end{cases} $$

where

$$\begin{aligned}& y_{0}^{l}=\sum_{i=1}^{N} \sum_{j=1}^{n} \min \bigl\{ {c_{ij}} {L_{i}}y_{j} ^{l},{c_{ij}} {U_{i}}y_{j}^{u} \bigr\} + \sum _{i=1}^{N} \min \{{c_{0i}} {L _{i}},{c_{0i}} {U_{i}}\}, \\& y_{0}^{u}=\sum_{i=1}^{N} \sum_{j=1}^{n} \max \bigl\{ {c_{ij}} {L_{i}}y_{j} ^{l},{c_{ij}} {U_{i}}y_{j}^{u} \bigr\} + \sum _{i=1}^{N} \max \{{c_{0i}} {L _{i}},{c_{0i}} {U_{i}}\}. \end{aligned}$$

Then problems (FP2) and (FP) are equivalent in the sense of the following result.

### Proposition 2.1

$y^{*}\in R^{n}$ *is a globally optimal solution for problem* (FP) *if and only if* $(y_{0}^{\ast }, {y}^{*},w ^{*})\in R^{n+N+1}$ *with* $y_{0}^{\ast }\in R$ *and* $w^{*}\in R^{N}$ *is a globally optimal solution for problem* (FP2), *where* $y_{0}^{\ast }= \sum_{i=1}^{N} w_{i}^{*}(c_{i}^{\top }y^{*}+c_{0i})$ *and* $w_{i}^{*}=\frac{1}{d_{i}({y}^{*})+d_{0i}}$ *for each* $i=1,\ldots ,N$.

### Proof

The proof of this result is obvious. □

From Proposition 2.1, notice that, in order to globally solve problem (FP), we may globally solve problem (FP2) instead. Moreover, it is easy to see that the global optimal values of problems (FP) and (FP2) are equal.

In addition, for convenience of the following discussion, let us denote $x=(y_{0},y,w)\in R^{n+N+1}$ with $y\in R^{n}$, $w\in R^{N}$. Then problem (FP2) can be rewritten as problem (P):

$$ \mbox{(P)}: \textstyle\begin{cases} \min & F(x)=x_{0} \\ \mbox{s.t.} & G_{k}(x)\leq 0, \quad k=0,\ldots ,m+N, \\ & x\in D^{0}, \end{cases} $$

where

$$ \textstyle\begin{array}{ll} D^{0}&= \{x\in R^{n+N+1} \mid x_{j}^{l} \leq x_{j}\leq x_{j}^{u}, j=0,1, \ldots ,n+N\} \\ &=\left \{ \textstyle\begin{array}{lll} x\in R^{n+N+1} \Bigg| \textstyle\begin{array}{lll} & x_{j}^{l}\triangleq y_{j}^{l}\leq x_{j}=y_{j}\leq x_{j}^{u}\triangleq y_{j}^{u}, &j=0,1,\ldots ,n, \\ & L_{j-n}\leq x_{j}=w_{j-n}\leq U_{j-n}, &j=n+1,\ldots ,n+N \end{array}\displaystyle \end{array}\displaystyle \right \} \end{array} $$

and $G_{k}(x)=G_{k}^{+}(x)-G_{k}^{-}(x)$ with $G_{k}^{+}(x)$, $G_{k} ^{-}(x)$ being increasing functions given by

$$ G_{k}^{+}(x)= \textstyle\begin{cases} \sum_{i=1}^{N} \sum_{j\in J_{i}^{+}} c_{ij}x_{j}x_{i+n}+\sum_{i\in I_{0}^{+}} c_{0i}x_{i+n}, &k=0, \\ \sum_{j\in T_{k}^{-}}(-d_{kj})x_{j}x_{k+n}+1, &k=1,\ldots ,N',\\ \sum_{j\in T_{k}^{-}}(-d_{kj})x_{j}x_{k+n}+(-d_{0k})x_{k+n}+1,&k=N'+1,\ldots ,N, \\ \sum_{j\in M_{k-N}^{+}}a_{k-N,j}x_{j}-b_{k-N}, & k=N+1,\ldots ,N+m, \end{cases} $$

and

$$ G_{k}^{-}(x)= \textstyle\begin{cases} \sum_{i=1}^{N} \sum_{j\in J_{i}^{-}} (-c_{ij})x _{j}x_{i+n}+\sum_{i\in I_{0}^{-}} (-c_{0i})x_{i+n}, &k=0, \\ \sum_{j\in T_{k}^{+}}d_{kj}x_{j}x_{k+n}+d_{0k}x_{k+n}, &k=1, \ldots ,N', \\ \sum_{j\in T_{k}^{+}}d_{kj}x_{j}x_{k+n}, & k=N'+1,\ldots ,N, \\ \sum_{j\in M_{k-N}^{-}}(-a_{k-N,j})x_{j}, & k=N+1,\ldots ,N+m. \end{cases} $$

Note that both problem (FP) and problem (P) are equivalent according to Proposition 2.1, hence for globally solving problem (FP), the algorithm to be presented concentrates on how to solve problem (P).

## Essential operations

For solving problem (P), we first give the following concept about an approximate optimal solution.

### Definition 3.1

For given $\varepsilon ,\eta \ge 0$, let

$$ D_{\varepsilon }= \bigl\{ x\in D^{0} \mid G_{k}(x)< \varepsilon , k=0,1,\ldots ,m+N \bigr\} , $$

a point $x\in D_{\varepsilon }$ is said to be an *ε*-feasible solution to problem (P). If an *ε*-feasible solution $\bar{x}=(\bar{x}_{0},\bar{x}_{1},\ldots ,\bar{x}_{n+N})$ to problem (P) satisfies

$$ \bar{x}_{0}\leq \min \{x_{0}\mid x\in D_{\varepsilon }\}+ \eta , $$

*x̄* is called an $(\varepsilon ,\eta)$-optimal solution to problem (P).

### Remark 3.1

All feasible solutions to problem (P) are *ε*-feasible. When $\eta =0$, an $(\varepsilon ,\eta)$-optimal solution is optimal over all *ε*-feasible solutions of problem (P). When $\eta >0$, an $(\varepsilon ,\eta)$-optimal solution is *η*-optimal for all *ε*-feasible solutions to problem (P).

For seeking an $(\varepsilon ,\eta)$-optimal solution of problem (P), a division and cut algorithm to be developed includes three essential operations: division operation, deleting operation, and reduction operation.

First, the division operation consists in a sequential box division of the original box $D^{0}$ following in an exhaustive subsection principle, such that any infinite nested sequence of division sets generated through the algorithm reduces to a singleton. This paper takes an adaptive division operation, which extends the standard bisection in the normally used exhaustive subsection principle. Second, by using overestimation of the constraints, the deleting operation consists in eliminating each subbox *D* generated by the division operation, in which there is no feasible solution. In addition, the reduction operation is used to reduce the size of the current partition set (referred to a node), aiming at tightening each subbox which contains the feasible portion currently still of interest.

For any box $D=[p,q]=\prod_{i=0}^{n+N}[p_{i},q_{i}]\subseteq D ^{0}$, for convenience, we will use the following functions throughout this paper:

$$\begin{aligned}& f_{k}^{i}(\alpha)=G_{k}^{+}(p)-G_{k}^{-} \bigl(q-\alpha (q_{i}-p_{i})e^{i}\bigr),\quad k=0,1,\dots ,m+N, \\& g_{k}^{i}(\beta)=G_{k}^{+} \bigl(p^{\prime }+\beta \bigl(q_{i}-p^{\prime }_{i} \bigr)e^{i}\bigr)-G_{k}^{-}(q), \quad k=0,1,\dots ,m+N, \end{aligned}$$

where $\alpha ,\beta \in (0,1),p_{i}\le p^{\prime }_{i}\le q_{i} $, and $e^{i}$ denotes the *i*th unit vector, i.e., a vector with 1 at the *i*th position and 0 everywhere else, $i=0,1,\dots , n+N$.

At a given stage of the proposed algorithm for problem (P), let *V* represent the best current objective function value to problem (P). Next, we will show these detailed operations.

### Deleting operation

In this subsection, we will give a suitable deleting operation, which offers a possibility to remove a subbox *D* of $D^{0}$ without feasibility. Toward this end, we take into account a subproblem of problem (P) over a given box $D=[p,q]=\prod_{i=0}^{n+N}[p_{i},q _{i}]\subseteq D^{0}$ as follows:

$$ \mathrm{P}(D): \textstyle\begin{cases} \min &x_{0}, \\ \mbox{s.t.} &\bar{F}(x)\triangleq \max \{G_{k}^{+}(x)-G_{k}^{-}(x)\mid k=0,1,\ldots ,m+N\}\leq 0, \\ & x=(x_{0},x_{1},\ldots ,x_{n+N})\in D. \end{cases} $$

Given $\eta >0$, for solving $\mathrm{P}(D)$, we need to seek out a feasible solution $\bar{x}=(\bar{x}_{0},\bar{x}_{1},\ldots , \bar{x}_{n+N}) \in D$ of $\mathrm{P}(D)$ such that $\bar{x}_{0}\leq V-\eta $, or to draw a conclusion that there exists no such *x̄*, where *V* is the best objective value to problem $\mathrm{P}(D)$ known so far. Obviously, if $p_{0}>V-\eta $, there exists no $\bar{x}\in D$ with $\bar{x}_{0}\leq V-\eta $. If $p_{0}\leq V-\eta $ and $G_{k}(p)\leq 0$ for each $k=0,\ldots ,m+N$, then update $\bar{x}=p$. Therefore, without loss of generality, in the following discussion we shall suppose that

3.1 $$ \bar{F}(p)>\varepsilon , \qquad p_{0}\leq V-\eta ,\qquad \bigl\{ x\in D^{0} \mid \bar{F}(x)< \varepsilon \bigr\} \neq \emptyset . $$

Clearly, if $\bar{F}(x)>\varepsilon $ for any $x\in D$, there exists no feasible solution on *D*, and so *D* is deleted for further discussion. However, since the judgement satisfying $\bar{F}(x)>\varepsilon $ is not easy, we introduce an auxiliary problem of $\mathrm{P}(D)$ as follows:

$$ \mathrm{Q}(D): \textstyle\begin{cases} \min & \bar{F}(x) \\ \mbox{s.t.}& x_{0}\leq V-\eta , \\ & x=(x_{0},x_{1},\ldots ,x_{n+N})\in D\subseteq D^{0}. \end{cases} $$

Observe that the objective and constraint functions are interchanged in $\mathrm{P}(D)$ and $\mathrm{Q}(D)$. Let $V(\mathrm{P}(D))$ and $V(\mathrm{Q}(D))$ be the optimal values of problems $\mathrm{P}(D)$ and $\mathrm{Q}(D)$, respectively. Then we give the following results about problems $\mathrm{P}(D)$ and $\mathrm{Q}(D)$.

#### Theorem 3.1

*Let* $\varepsilon >0$, $\eta >0$ *be given*, *and let* *D* *be a box with* $D\subseteq D^{0}$. *We have the following result*:

(i) *A feasible solution to* $\mathrm{Q}(D)$ *satisfying* $\bar{F}(\hat{x})< \varepsilon $ *is an* *ε*-*feasible solution of* $\mathrm{P}(D)$ *with* $\hat{x}_{0}\leq V-\eta $.
(ii) *If* $V(\mathrm{Q}(D^{0}))>0$, *consider the following two cases*: (a) *problem P*($D^{0}$) *has no feasible solution if* $V=x_{0}^{u}+\eta $, *and* (b) *an* *ε*-*feasible solution* $\tilde{x}=(\tilde{x}_{0},\tilde{x} _{1},\ldots ,\tilde{x}_{n+N})$ *of* $\mathrm{Q}(D^{0})$ *is an* $(\varepsilon , \eta)$-*optimal solution of* $\mathrm{P}(D^{0})$ *if* $V=\tilde{x}_{0}$.

#### Proof

(i) This result is obvious, and here it is omitted.

(ii) By utilizing the assumption $V(\mathrm{Q}(D^{0}))>0$, i.e.,

3.2 $$ \min \bigl\{ \bar{F}(x)\mid x_{0}\leq V-\eta ,x\in D^{0} \bigr\} >0, $$

we have the following conclusions:

- If $V=x^{u}_{0}+\eta $, by (3.2) we get $\{x\mid \bar{F}(x) \le 0, x\in D^{0}\}=\emptyset $, which implies that problem $\mathrm{P}(D)$ has no feasible solution.
- If $V=\tilde{x}_{0}$, from (3.2) it is easy to see that
  $$ x_{0}>V-\eta =\tilde{x}_{0}-\eta , $$
  for any $x=(x_{0},x_{1},\ldots ,x_{n+N})\in \{x\mid \bar{F}(x)\leq 0 < \varepsilon , x\in D^{0}\}$, which means that
  $$ \min \bigl\{ x_{0}\mid G_{k}(x)< \varepsilon , k=0,1, \ldots ,m+N, x \in D^{0} \bigr\} \geq \tilde{x}_{0}-\eta . $$

Consequently, *x̃* is an $(\varepsilon ,\eta)$-optimal solution of $\mathrm{P}(D)$, and this accomplishes the proof. □

Theorem 3.1 illustrates that by utilizing problem $\mathrm{Q}(D)$ one can know whether or not there exists a feasible solution $\hat{x}=(\hat{x}_{0}, \hat{x}_{1},\ldots ,\hat{x}_{n+N})$ of $\mathrm{P}(D)$ with improving the current objective function value *V*, i.e., $\hat{x}_{0}\leq V-\eta $. Further, if $V(\mathrm{Q}(D^{0}))\ge \varepsilon >0$, then an $(\varepsilon ,\eta)$-optimal solution of $\mathrm{P}(D^{0})$ can be obtained, or it can be confirmed that problem $\mathrm{P}(D^{0})$ has no feasible solution.

Additionally, if $V(\mathrm{Q}(D))>\varepsilon $, it is easy to see that a fathomed box *D* cannot contain the feasible solutions for problem (P). Thus, *D* is excluded from further consideration by the search. Unfortunately, solving problem $\mathrm{Q}(D)$ may be as difficult as solving the original problem $\mathrm{P}(D)$. Hence, a lower bound $\operatorname{LB}(D)$ of $V(\mathrm{Q}(D))$ is required for eliminating the part of $D^{0}$ which does not contain the solution to problem (P). Clearly, if $\operatorname{LB}(D)\geq \varepsilon $, *D* is excluded from further consideration by the search. Since $G_{k}^{+}(x)$ and $G_{k}^{-}(x)$ are all increasing, an apparent lower bound is

$$ \underline{\operatorname{LB}}(D)=\max_{k=0,1,\ldots ,m+N} \bigl\{ G_{k}^{+}(p)-G_{k}^{-}(q)\bigr\} . $$

Although quite straightforward, the bound is sufficient to confirm convergence of the algorithm, as we will see immediately. For strengthening the computational validity in solving problem $\mathrm{P}(D)$, some tighter lower bounds can be obtained by utilizing the following Theorem 3.2. Especially, what is more important is that such tighter lower bounds can be gained only by simple arithmetic computations, which is different from the ones in the usual branch and bound methods for solving convex or linear programs [5, 6, 11, 12, 14].

#### Theorem 3.2

*For any* $D=[p,q]=\prod_{i=0}^{n+N}[p _{i},q_{i}]\subseteq D^{0}$, *under assumption* (3.1), *let*

$$ \alpha = \textstyle\begin{cases} \frac{q_{0}-V+\eta }{q_{0}-p_{0}}, & \textit{if } q_{0}>V-\eta, \\ 0, & \textit{otherwise}. \end{cases} $$

*Then a lower bound* $\operatorname{LB}(D)$ *satisfying* $\operatorname{LB}(D)\leq V(Q(D))$ *for problem* $\mathrm{Q}(D)$ *is given by*

$$ \operatorname{LB}(D)=\min_{i=0,1,\ldots ,n+N} \max_{k=0,1,\ldots ,m+N} \bigl\{ f_{k}^{i}( \alpha) \bigr\} . $$

#### Proof

(i) If $\alpha =0 $, this result is obvious.

(ii) By the assumption it holds that $p_{0}\leq V-\eta < q_{0}$. Then we get $0<\alpha <1$, and there exists $\tilde{x}=q-\alpha (q-p)$ so that $\tilde{x}_{0}=V-\eta $ with $\tilde{x}_{0}=q_{0}-\alpha (q_{0}-p _{0})$. Thus, for each $\hat{x}=q-\beta (q-p)$ with $\beta <\alpha $, it is easy to find that $\hat{x}_{0}>\tilde{x}_{0}=V-\eta $, that is, for each $x>\tilde{x}$, there exists $\hat{x}=q-\beta (q-p)$ with $\beta <\alpha $ such that $x\geq \hat{x}$, and so it holds that $x_{0}\geq \hat{x}_{0}>V-\eta $. Now, let us denote

$$ \Omega^{i}= \bigl\{ x\in [p,q]\mid p_{i}\leq x_{i}\leq \tilde{x_{i}} \bigr\} , $$

then we can acquire that

$$\begin{aligned} \bigl\{ x\in [p,q]\mid x_{0}\leq V-\eta \bigr\} &\subset [p,q] \setminus \{x\mid\tilde{x}< x\leq q\} \\ &\ =[p,q]\Bigm\backslash \bigcap_{i=0}^{n+N}{ \bigl\{ x\in [p,q]\mid \tilde{x_{i}}< x _{i} \bigr\} } \\ & = \bigcup_{i=0}^{n+N} \bigl\{ x\in [p,q]\mid p_{i} \leq x_{i} \leq \tilde{x_{i}} \bigr\} \\ & = \bigcup_{i=0}^{n+N}{ \Omega^{i}}. \end{aligned}$$

Let $\operatorname{LB}(\Omega^{i})= \max_{k=0,1,\ldots ,m+N}\{f_{k}^{i}( \alpha)\}$ and $\operatorname{LB}(D)=\min \{\operatorname{LB}(\Omega^{i})\mid i=0,1,\ldots ,n+N\}$. Obviously, we get $\operatorname{LB}(\Omega^{i})\leq \min \{\bar{F}(x) \mid x\in \bigcup_{i=0}^{n+N}{\Omega^{i}}\}$,

$$ \operatorname{LB}(D) \leq \min \Biggl\{ \bar{F}(x)\Bigm| x\in \bigcup _{i=0}^{n+N} {\Omega^{i}} \Biggr\} \leq \min \bigl\{ \bar{F}(x)\mid x_{0}\leq V-\eta ,x\in [p,q] \bigr\} . $$

Thus, the proof is complete. □

Notice that $\operatorname{LB}(D)$ in Theorem 3.2 satisfies

3.3 $$ \min \bigl\{ \bar{F}(x)\mid x_{0}\leq V-\eta ,x\in D^{0} \bigr\} \geq \operatorname{LB}(D)\geq\max_{k=0,1,\ldots ,m+N} \bigl\{ G_{k}^{+}(p)-G_{k}^{-}(q) \bigr\} . $$

### Adaptive division

The division operation repeatedly subdivides an $(n+N+1)$-dimensional box $D^{0}$ into $(n+N+1)$-dimensional subboxes. This operation helps the algorithm confirm the position of a global optimal solution in $D^{0}$ for problem (P). Throughout this algorithm, we take a new adaptive subdivision principle as follows.

#### Adaptive subdivision

For given $\eta >0$, consider any box $D=[p,q]=\{x\in R^{n+N+1}|p_{i} \le x_{i}\le q_{i},i=0,1,\ldots ,n+N\}\subseteq D^{0}$.

(i) If $q_{0}>V-\eta $, then let $\alpha =\frac{q_{0}-V+\eta }{q_{0}-p _{0}}$; otherwise let $\alpha =0$.

(ii) Denote $t=\mathrm{{argmax}}\{q_{i}-p_{i}\mid i=0,1,\dots ,n+N\}$. Let $u_{t}=p_{t}$ and $v_{t}=q_{t}-\alpha (q_{t}-p_{t})$. Set $\bar{x} _{t}=(u_{t}+v_{t})/2$.

(iii) By using $\bar{x}_{t}$, let us denote

$$ D_{1}= \bigl\{ x\in R^{n+N+1}\mid p_{i}\leq x_{i}\leq q_{i},i\neq t, p_{t} \leq x_{t}\leq \bar{x}_{t}, i=0,1,\dots ,n+N \bigr\} , $$

and

$$ D_{2}= \bigl\{ x\in R^{n+N+1}\mid p_{i}\leq x_{i}\leq q_{i},i\neq t, \bar{x} _{t}\leq x_{t}\leq q_{t} , i=0,1,\dots ,n+N \bigr\} . $$

Based on the above division operation, *D* is divided into two new boxes $D_{1}$ and $D_{2}$. Especially, when $\alpha =0$, the adaptive subdivision simply reduces to the standard bisection. As we will see from numerical experiments in Sect. 5, the adaptive subdivision is superior to the standard bisection. Moreover, the subdivision can confirm the convergence of the algorithm, and we have the following results.

#### Theorem 3.3

*Suppose that the above adaptive division operation is infinite*, *then it generates a nested sequence* $\{D^{s _{t}}\}$ *of partition sets* $\{D^{s}\}$ *generated by the adaptive division operation*, *so that* $\operatorname{LB}(D^{s_{t}})\rightarrow V(\mathrm{Q}(D^{0}))$ *as* $t\rightarrow +\infty $.

#### Proof

By the adaptive division operation, for each box $D^{s}=[p^{s},q^{s}]\subseteq D^{0}$, we can acquire the points $u^{s}, v^{s}(i)\in D^{s}$ ($i=0,1,\dots ,n+N$) satisfying

$$ u^{s}=p^{s},\qquad v^{s}(i)=q^{s}-\alpha^{s} \bigl(q^{s}_{i}-p^{s}_{i}\bigr)e^{i} \quad \mbox{with } \alpha^{s}=0 \mbox{ or } \alpha^{s}=\frac{q^{s}_{0}-V+ \eta }{q^{s}_{0}-p^{s}_{0}} . $$

From Theorem 3.2, we can obtain

$$ \operatorname{LB} \bigl(D^{s} \bigr)=\min_{i=0,1,\ldots ,n+N}\max _{k=0,1,\ldots ,m+N} \bigl\{ G_{k}^{+} \bigl(u ^{s} \bigr)- G_{k}^{-} \bigl(v^{s}(i) \bigr) \bigr\} . $$

According to Ref. [18], this adaptive division ensures the existence of an infinite subsequence $\{s_{t}\}$ with $D^{s_{t}+1}\subseteq D^{s _{t}}$ and $\operatorname{LB}(D^{s_{t}})\le V(Q(D^{0}))$ for each *t*, so that for each $i=0,1,\dots ,n+N$,

$$\begin{aligned}& v^{s_{t}}(i)-u^{s_{t}}\rightarrow 0,\quad \mbox{as } t\rightarrow +\infty , \\& \lim_{t \to +\infty }v^{s_{t}}(i)=\lim_{t \to +\infty }u^{s_{t}}=\hat{u}\in D^{0}. \end{aligned}$$

Thus we can obtain that

$$ \lim_{t \to +\infty }\operatorname{LB} \bigl(D^{s_{t}} \bigr)=\min _{i=0,1,\ldots ,n+N} \max_{k=0,1,\ldots ,m+N} \bigl\{ G_{k}^{+}( \hat{u})-G_{k}^{-}(\hat{u}) \bigr\} = \bar{F}(\hat{u}). $$

In addition, by assumption (3.1), since $u_{0}^{s_{t}}=p_{0}^{s_{t}} \leq V-\eta $, it holds that

$$ \lim_{t \to +\infty }u_{0}^{s_{t}}= \hat{u}_{0}\le V-\eta , $$

which implies that *û* is feasible to Q($D^{0}$), then we get

$$ \operatorname{LB} \bigl(D^{s_{t}} \bigr)\le \min \bigl\{ \bar{F}(x)\mid u_{0} \leq V-\eta ,u\in D^{0} \bigr\} \le \bar{F}(\hat{u}). $$

Consequently, the limitation $\operatorname{LB}(D^{s_{t}})\rightarrow V(Q(D^{0}))$ $(t \rightarrow +\infty)$ holds and then we accomplish the proof. □

### Reduction operation

At a given stage of the proposed algorithm for problem (P), let $D=[p,q]\subseteq D^{0}$ be a box generated by the division operation and still of interest. Clearly, the smaller this box *D* is, the closer the feasible solution will be to the $(\varepsilon ,\eta)$-optimal solution to problem (P). Hence, to effectively tighten the variable bounds in a particular node, a valid range reduction strategy is introduced by overestimation of the constraints, and by applying the monotonic decompositions to problem (P). Based on the above discussion, for any box $D=[p,q]\subseteq D^{0}$ generated by the division operation and still of interest, we intend to identify whether or not the box *D* contains a feasible solution *x̂* of $\mathrm{P}(D)$ such that $\hat{x}_{0}\leq V-\eta $. Consequently, seeking such a point *x̂* can be confined to the set $\hat{D}\cap D$, where

$$ \hat{D}= \bigl\{ x\mid x_{0}\leq V-\eta , G_{k}^{+}(x)-G_{k}^{-}(x)< \varepsilon , k=0,1,\dots ,m+N \bigr\} . $$

The reduction operation is based on special cuts that exploit the monotonic structure of problem (P), and it aims at substituting the box $D=[p,q]$ with a smaller box $D'$ without losing any valid point $x\in \hat{D}\cap D$, i.e.,

3.4 $$ \hat{D}\cap D'=\hat{D}\cap D. $$

This will suppress the fast growth of the branching tree in the division operation for seeking the $(\varepsilon ,\eta)$-optimal solution of problem (P).

For any $D=[p,q]=\prod_{i=0}^{n+N}[p_{i},q_{i}]\subseteq D^{0}$ generated by the division operation, the box $D'$ satisfying condition (3.4) is denoted by $R[p,q]$. To recognize how $R[p,q]$ is acquired, we first demand to know the parameters *γ*, ${\alpha }_{k}^{i}$, and ${\beta }_{k}^{i}$ computed for each $k=0,1,\dots ,m+N$, $i=0,1, \dots ,n+N$ by utilizing the following rules.

*Rule* (i): Given the box $[p,q]\subseteq D^{0}$, if $f_{k}^{i}(1) >\varepsilon $, let ${\alpha }_{k}^{i}$ be the solution to the equation $f_{k}^{i}({\alpha }_{k}^{i})=\varepsilon $ about the univariate $\alpha_{k}^{i}$; otherwise let ${\alpha }_{k}^{i}=1$.

*Rule* (ii): For given boxes $D=[p,q]$ and $D'=[\bar{p},q]$ with $D'\subseteq D\subseteq D^{0}$, if $g_{k}^{i}(1) >\varepsilon $, one can solve the univariate equation $g_{k}^{i}({\beta }_{k}^{i})= \varepsilon $ to obtain ${\beta }_{k}^{i}$; otherwise let ${\beta } _{k}^{i}=1$. If $\bar{p}_{0}< V-\eta < q_{0}$, then set $\gamma =\frac{V- \eta -\bar{p}_{0}}{q_{0}-\bar{p}_{0}}$; otherwise let $\gamma =1$.

Notice that it is easy to get ${\alpha }_{k}^{i}$ and $\beta_{k}^{i}$, since the univariate functions $f_{k}^{i}(\lambda)$ and $g_{k}^{i}( \mu)$ are strictly monotonic in Rules (i) and (ii).

According to Rules (i) and (ii), let us denote

3.5 $$\begin{aligned}& \hat{\alpha }^{i}=\min_{k=0,1,\ldots ,m+N} \bigl\{ {\alpha }_{k}^{i} \bigr\} , \\& \hat{\beta }^{i}=\min_{k=0,1,\ldots ,m+N} \bigl\{ {\beta }_{k}^{i}, \gamma\bigr\} , \quad i=0,1,\ldots ,n+N, \end{aligned}$$

then $R[p,q]$ can be obtained by Theorems 3.4 and 3.5.

#### Theorem 3.4

*Given the box* $D=[p,q]=\prod_{i=0} ^{n+N}[p_{i},q_{i}]\subseteq D^{0}$, *it holds that*

(i) *If* $p_{0}\leq V-\eta $ *and* $\bar{F}(p)<\varepsilon $, *then* $R[p,q]=[p,p]$, *and*
(ii) *If* $p_{0}>V-\eta $ *or* $\max \{f_{k}^{i}(0) | k=0,1,\dots ,m+N \}> \varepsilon $ *holds for some* $i\in \{0,1,\ldots ,n+N\}$, *then* $R[p,q]=\emptyset $.

#### Proof

(i) The proof of this result is easy, here it is omitted.

(ii) The former of the conclusion is apparent, we only need to give the proof of the latter.

If there exists some $i\in \{0,1,\ldots ,n+N\}$ so that $\max \{f_{k} ^{i}(0) | k=0,1,\dots ,m+N\}> \varepsilon $, we can obtain

$$ \bar{F}(x)>\max_{k=0,1,\dots ,m+N} \bigl\{ f_{k}^{i}(0)\bigr\} >\varepsilon \quad \mbox{for each } x\in [p,q]. $$

Therefore, we can acquire $R[p,q]=\emptyset $, and this accomplishes the proof. □

#### Theorem 3.5

*For any* $D=[p,q]\subseteq D^{0}$, *under Rule* (i) *and* (3.5) *and the assumption*

$$ p_{0}\leq V-\eta \quad \textit{ and }\quad \max_{k=0,1,\dots ,m+N} \bigl\{ f_{k}^{i}(0) \bigr\} < \varepsilon \quad \textit{for each } i=0,1,\ldots ,n+N, $$

*let* ${\hat{p}}=q-\sum_{i=0}^{n+N}\hat{\alpha }^{i}(q_{i}-p_{i})e ^{i}$. *If the box* $[\hat{p},q]$ *satisfies the assumption of Theorem *3.4, *then* $R[p,q]=[\hat{p},\hat{p}]$ *or* $R[p,q]=\emptyset $. *Otherwise*, $R[p,q]=[\hat{p},\hat{q}]$, *where* $\hat{q}=\hat{p}-\sum_{i=0}^{n+N}\hat{\beta }^{i}(q_{i}-p_{i})e^{i}$ *with respect to Rule* (ii) *and* (3.5).

#### Proof

For any given $x=(x_{0},\ldots ,x_{n+N})^{T}\in [p,q]$, we first confirm that $x\geq \hat{p}$.

Assume that $x\ngeq \hat{p}$, that is to say, there exists some $i\in \{0,1,\ldots ,n+N\}$ such that

3.6 $$ x_{i}< \hat{p}_{i}=q_{i}-\hat{\alpha }^{i}(q_{i}-p_{i}) \quad \mbox{i.e. } x_{i}=q_{i}-\alpha (q_{i}-p_{i}) \mbox{ with } \alpha > \hat{\alpha }^{i}. $$

Then we discuss as follows:

If $\hat{\alpha }^{i}=1$, we can obtain $x_{i}<\hat{p}_{i}=q_{i}- \hat{\alpha }^{i}(q_{i}-p_{i})=p_{i}$ from (3.6), contradicting $x\in [p,q]$, then $x\geq \hat{p}$.

If $\hat{\alpha }^{i}\in (0,1)$, from Rule (i) and the definition of $\hat{\alpha }^{i}$, there must exist some *k* such that $f_{k}^{i}( \hat{\alpha }^{i})=\varepsilon $, i.e.,

3.7 $$ G_{k}^{+}(p)-G_{k}^{-} \bigl(q-\hat{ \alpha }^{i}(q_{i}-p_{i})e^{i} \bigr)= \varepsilon. $$

In addition, through Rule (i) and the definition of $G_{k}^{-}(x)$, it holds from (3.6) and (3.7) that

$$ G_{k}^{-} \bigl(q-(q_{i}-x_{i})e^{i} \bigr)= G_{k}^{-} \bigl(q-\alpha (q_{i}-p_{i})e ^{i} \bigr)< G_{k}^{-} \bigl(q-\hat{\alpha }^{i}(q_{i}-p_{i})e^{i} \bigr)=G_{k}^{+}(p)- \varepsilon . $$

Consequently,

$$ G_{k}^{-}(x)\leq G_{k}^{-} \bigl(q-(q_{i}-x_{i})e^{i} \bigr)< G_{k}^{+}(p)- \varepsilon \leq G_{k}^{+}(x)-\varepsilon , $$

contradicting $G_{k}^{+}(x)-G_{k}^{-}(x)\leq \varepsilon $. According to the above discussions, we have demonstrated that $x\geq \hat{p}$, i.e., $x\in [\hat{p},q]$. Next we will show that $x\leq \hat{q}$.

Assume that $x\nleq \hat{q}$, then there exists some *i* such that

3.8 $$ x_{i}>\hat{q}_{i}=\hat{p}_{i}+\hat{\beta }^{i}(q_{i}-\hat{p}_{i}),\quad \mbox{i.e. } x_{i}=\hat{p}_{i}+\beta (q_{i}- \hat{p}_{i}) \mbox{ with } \beta >\hat{\beta }^{i}. $$

We discuss as follows:

If $\hat{\beta }^{i}=1$, from (3.8) we can acquire $x_{i}>\hat{q} _{i}=\hat{p}_{i}+\hat{\alpha }^{i}(q_{i}-\hat{p}_{i})=q_{i}$, contradicting $x\in [p,q]$.

If $\hat{\beta }^{i}\in (0,1)$, from Rules (i) and (ii) and the definition of $\hat{\beta }^{i}$, we can obtain $g_{k}^{i}( \hat{\beta }^{i})=\varepsilon $, i.e.,

3.9 $$ G_{k}^{+} \bigl(\hat{p}_{i}+\hat{\beta }^{i}(q_{i}-\hat{p}_{i})e^{i} \bigr)-G_{k}^{-}(q)=\varepsilon , $$

or

3.10 $$ \hat{p}_{0}+\hat{\beta }^{0}(q_{0}- \hat{p}_{0})=V-\eta . $$

Suppose that (3.9) holds, due to Rule (ii) and the definition of $G_{k}^{+}(x)$, by (3.8) and (3.9) it follows that

$$ G_{k}^{+} \bigl(\hat{p}+(x_{i}- \hat{p}_{i})e^{i} \bigr)=G_{k}^{+} \bigl( \hat{p}+\beta (q _{i}-\hat{p}_{i})e^{i} \bigr)> G_{k}^{+} \bigl(\hat{p}+\hat{\beta }^{i}(q_{i}- \hat{p}_{i})e^{i} \bigr)=G_{k}^{-}(q)+ \varepsilon ; $$

therefore,

$$ G_{k}^{+}(x)\geq G_{k}^{+} \bigl( \hat{p}+(x_{i}-\hat{p}_{i})e^{i} \bigr)>G_{k} ^{-}(q)+\varepsilon \geq G_{k}^{-}(x)+ \varepsilon \quad \mbox{with } x _{i}=\hat{p}_{i}+\beta (q_{i}-\hat{p}_{i}), $$

which contradicts $G_{k}^{+}(x)-G_{k}^{-}(x)\leq \varepsilon $.

Suppose that (3.10) holds, from (3.8), (3.10), and Rule (ii), we can draw a conclusion that

$$ x_{0}=\hat{p}_{0}+\beta (q_{0}- \hat{p}_{0})>\hat{p}_{0}+\hat{\beta } ^{0}(q_{0}- \hat{p}_{0})=V-\eta , $$

which contradicts $x_{0}\leq V-\eta $. According to the above discussions, we can acquire $x\leq \hat{q}$, i.e., $x\in [\hat{p},\hat{q}]$, and the proof is complete. □

From Theorem 3.5, by Rules (i) and (ii) the main computational effort for deriving $R[p,q]$ is to solve some univariate equations about the variables ${\hat{\alpha }}_{k}^{i}$ and $\hat{\beta }_{k}^{i}$, which is easy to solve, for example, by using the bisection approach. What is more, as seen below, the cost of main computation in the proposed algorithm is also to form $R[p,q]$.

## Algorithm and its convergence

According to the above discussions, the proposed algorithm is shown as follows.

### Algorithm statement

*Step* (0) Given tolerances $\varepsilon ,\eta >0$, if there is no known feasible solution at present, set $V=x^{u}_{0}$ with $D^{0}=[x^{l},x^{u}]$; otherwise let $x^{\ast }$ be the best feasible solution of problem (P), and set $V=x_{0}^{\ast }$. Let $M_{0}=\{D ^{0}\}$, $N_{0}=\emptyset , t=0$.

*Step* (1) For each $D=[p,q]=\prod_{i=0}^{n+N}[p_{i},q _{i}] \in M_{t}$, compute $R[p,q]$ according to Theorems 3.4 and 3.5. Then:

- If $R[p,q]=\emptyset $, eliminate *D*.
- If $R[p,q]=[p,p]$, discard *D* and update $x^{\ast }=p$, $V=p_{0}$.
- If $R[p,q]\neq \emptyset $, set $D=R[p,q]$ and compute the lower bound $\operatorname{LB}(D)$ by Theorem 3.5. If $\operatorname{LB}(D)>\varepsilon $, delete *D*.

*Step* (2) Denote $\bar{M_{t}}$ to be the collection of boxes that results from $M_{t}$ after accomplishment of Step (1), and set $\bar{N_{t}}=N_{t}\cup \bar{M_{t}}$.

- If $\bar{N_{t}}=\emptyset $, stop: (i) if $V=x^{u}_{0}$, problem (P) is infeasible; (ii) if $V=x_{0}^{\ast }$, $x^{\ast }$ is an $(\varepsilon ,\eta)$-optimal solution of problem (P).
- If $\bar{N_{t}} \neq \emptyset $, Theorem 3.4 is applied to each $D\in \bar{N_{t}}$, then eliminate *D* and update $x^{\ast }=p$, $V=p_{0}$ if necessary.

*Step* (3) Denote $\bar{N_{t}}$ to be the collection of boxes after accomplishment of Step (2). Choose $D^{t}=[p^{t},q^{t}] \in {\mathrm{{argmin}}}\{\operatorname{LB}(D)\mid D\in \bar{N_{t}}\}$, and denote $\operatorname{LB}_{t}=\operatorname{LB}(\bar{N _{t}})$. If $\operatorname{LB}_{t}>0$, then terminate: the conclusion is the same as situation (a) of Step (2); otherwise go to Step (4).

*Step* (4) If $q_{0}^{t}>V-\eta $, let $s^{t}=p^{t}+\alpha_{t}(q ^{t}-p^{t})$ with $\alpha_{t}=\frac{q_{0}-V+\eta }{q_{0}-p_{0}}$; otherwise set $s^{t}=p^{t}$ or $s^{t}=(p^{t}+q^{t})/2$. If $\bar{F}(s ^{t})<\varepsilon $, update $x^{\ast }=s^{t}$, $V=s_{0}^{t}$.

*Step* (5) Divide $D^{t}$ into two subboxes by the adaptive division, and set $M_{t+1}$ to be the collection of these two subboxes of $D^{t}$. Let $N_{t+1}=\bar{N_{t}}\backslash \{D^{t}\}$. Set $t=t+1$, and return to Step (1).

### Remark

By utilizing a local solver in Step (4) of the proposed algorithm, instead of evaluating one point, we may acquire a point with the better objective function value *V*, and the iteration count of the algorithm may be reduced. However, because the computational cost increases rapidly with the quality of the objective value *V*, the running time is not always decreasing. So a trade-off must be made, practically just one evaluating point as in the above algorithm is used. Moreover, to implement the algorithm, all that is required is the ability to solve univariate equations with monotonicity and to execute simple algebraic steps.

The convergence of the algorithm is shown by the following theorem.

### Theorem 4.1

*For given tolerances* $\varepsilon ,\eta >0$, *the above algorithm always terminates after finitely many iterations*, *obtaining an* $(\varepsilon ,\eta)$-*optimal solution of problem* (P), *or a demonstration that the problem is infeasible*.

### Proof

Since any feasible solution $x=(x_{0},x_{1},\dots ,x _{n+N})$ to problem (P) with $x_{0}\leq V-\eta $ must exist in some box $D\in \bar{N_{t}}$, case (a) of Step (2) implies that there exists no such solution *x*. In addition, if $\operatorname{LB}_{t}>0$ occurs in Step (3), then we can obtain $V(\operatorname{MQ}(D))>0$. Consequently, by Theorem 3.5 the conclusion is true if one of the following cases occurs:

$$ \bar{N_{t}}=\emptyset \quad \mbox{or}\quad \operatorname{LB}_{t} >0, $$

that is to say, for extremely large *t* Steps (4) and (5) cannot appear. It remains to illustrate that either $\bar{N_{t}}=\emptyset $ or $\operatorname{LB}_{t} >0$ must take place for extremely large *t*.

By contradiction, assume that the algorithm is infinite. Because each appearance of Step (4) reduces the current best objective function value $V=x_{0}^{\ast }$ at least by $\eta >0$ if $\bar{F}(s^{t})< \varepsilon $, this conflicts with the fact that $x_{0}$ is bounded below if Step (4) takes place infinitely. In other words, $\bar{F}(s^{t})< \varepsilon $ in Step (4) cannot occur infinitely often. Therefore, for all *t* extremely large, $x^{\ast }$ is unaltered, and $\bar{F}(s^{t})\ge \varepsilon$ while $\operatorname{LB}_{t}\leq 0$, and then Step (5) must be performed infinitely. By the division operation of the algorithm, as $t\rightarrow +\infty $, $q^{t}-p^{t} \rightarrow 0$, we have

$$ \lim_{t \to +\infty }p^{t}=\lim_{t \to +\infty }q^{t}= \lim_{t \to +\infty }s^{t}=\hat{x}\in D^{0}. $$

According to (3.5), it holds that

$$ 0\geq \operatorname{LB}_{t}\geq \max_{k=0,1,\ldots ,m+N} \bigl\{ G_{k}^{+} \bigl(p^{t} \bigr)-G_{k}^{-} \bigl(q ^{t} \bigr) \bigr\} , $$

thus, it follows that

$$ 0\geq \lim_{t \to +\infty }\operatorname{LB}_{t}\geq \max _{k=0,1,\ldots ,m+N} \bigl\{ G_{k} ^{+}( \hat{x})-G_{k}^{-}(\hat{x}) \bigr\} =\bar{F}(\hat{x}), $$

which conflicts with $\lim_{t \to +\infty }\bar{F}(s^{t})=\bar{F}( \hat{x})\ge \varepsilon >0$. Consequently, the algorithm must be finite, and the proof is finished. □

## Numerical experiments

**Figure 1:**
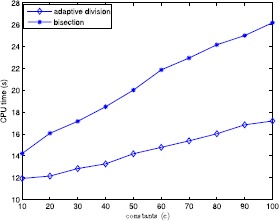
Average computational time in seconds when $n=3$, $N=50$ and $\varepsilon =0.05$

**Figure 2:**
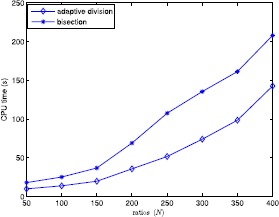
Average computational time in seconds when $n=3$ and $\varepsilon =0.05$

**Figure 3:**
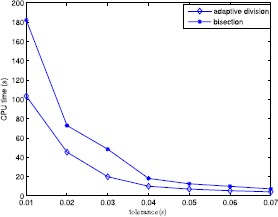
Average computational time in seconds when $n=3$ and $N=50$

**Figure 4:**
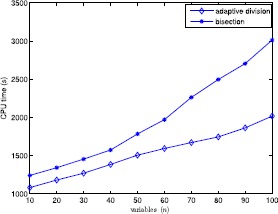
Average computational time in seconds when $N=50$ and $\varepsilon =0.05$

In this section, to verify the performance of the proposed algorithm, we give the computational results of six test examples and randomly generated problems. All tests are carried out on an Intel 5 CPU 2.33 GHz with 2 GB memory microcomputer and the algorithm is encoded in MATLAB. The numerical results are shown in Tables 1–2 and Figs. 1–4 to illustrate the feasibility and validity of the proposed algorithm. We first describe several simple examples in order to compare them with Refs. [4, 16], and the corresponding computational results are illustrated in Table 1. Additionally, we choose other problems randomly generated to test further the proposed algorithm, and the numerical results are shown in Figs. 1–4 and Table 2. As seen in the proposed algorithm, the main computational effort is to solve some univariate and monotonic equations to obtain ${\hat{\alpha }}_{k}^{i}$ and $\hat{\beta }_{k}^{i}$ required in the numerical experiments.

**Table 1:** Computational results for Examples 1–6

Ex.	Ref.	Solution	*ε*	*η*	Optimum	Iter.	$L_{\max}$	Time(s)
1	[ours]	(0.0000, 1.66666667, 0.0000)	10^−3^	10^−6^	3.710919	8	4	0.1830
	[4]	(0.0000, 0.625, 1.875)	10^−4^		4.0000	58	18	2.968694
	[17]	(1.1111, 1.36577e−05, 1.35168e−05)	10^−9^					
2	[ours]	(5.0000, 0.0000,0.0000)	10^−3^	10^−6^	2.861905	16	8	0.1250
	[4]	(0, 3.3333, 0)	10^−4^		3.0029	80	64	8.566259
3	[ours]	(1.5000, 1.5000)	10^−3^	10^−6^	4.9125874	56	14	1.0870
	[4]	(3.0000, 4.0000)	10^−4^		5	32	32	1.089285
	[17]	(3.0000, 4.0000)	10^−6^					
4	[ours]	(1.1111, 0.0000, 0.0000)	10^−2^	10^−5^	−4.090703	185	55	3.2510
	[16]	(1.0715, 0, 0)	10^−6^		−4.087412	17		
5	[ours]	(0.0000, 0.3333, 0.0000)	10^−3^	10^−6^	−3.0029	17	3	0.1290
	[16]	(0, 0.33329, 0)	10^−6^		−3.000042	30		
6	[ours]	(1.0000, 0.0000)	10^−3^	10^−6^	1.428571	6	2	0.0470
	[16]	(1.0000, 0.0000)	10^−6^		1.428571	10

**Table 2:** Average performances of adaptive division and bisection when $n=3$ and $\varepsilon =0.05$

	*N*	600	800	1000	1200	1500
Adaptive division	CPU time (s)	305.709	479.106	563.286	754.851	1009.027
Bisection	CPU time (s)	386.527	573.421	769.683	971.858	1227.389

The notation in Table 1 is as follows:

• Ref.: reference;

• Iter.: the number of the algorithm iterations;

• Time (s): CPU seconds required for solving a problem;

• $L_{\max}$: the maximal number of the active node necessary.

### Example 1

(see [4, 17])

$$\begin{aligned} &\min \quad \frac{4x_{1}+3x_{2}+3x_{3}+50}{3x_{2}+3x_{3}+50}+\frac{3x_{1}+4x_{3}+50}{4x_{1}+4x_{2}+5x_{3}+50} \\ &\qquad \qquad {}+\frac{x_{1}+2x_{2}+4x_{3}+50}{x_{1}+5x _{2}+5x_{3}+50}+ \frac{x_{1}+2x_{2}+4x_{3}+50}{5x_{2}+4x_{3}+50} \\ &\mbox{s.t.}\quad\quad 2x_{1}+x_{2}+5x_{3} \leq 10, \\ & \hphantom{\mbox{s.t.}\qquad}x_{1}+6x_{2}+2x_{3} \leq 10, \\ & \hphantom{\mbox{s.t.}\qquad}9x_{1}+7x_{2}+3x_{3} \geq 10, \\ & \hphantom{\mbox{s.t.}\qquad}x_{1},x_{2},x_{3} \geq 0. \end{aligned}$$

### Example 2

(see [4])

$$\begin{aligned} &\min\quad {\frac{3x_{1}+5x_{2}+3x_{3}+50}{3x_{1}+4x_{2}+5x_{3}+50}+ \frac{3x_{1}+4x_{2}+50}{4x_{1}+3x_{2}+2x_{3}+50}+\frac{4x_{1}+2x_{2}+4x_{3}+50}{5x _{1}+4x_{2}+3x_{3}+50}} \\ &\mbox{s.t.}\quad\quad 2x_{1}+x_{2}+5x_{3} \leq 10, \\ & \hphantom{\mbox{s.t.}\qquad}x_{1}+6x_{2}+2x_{3} \leq 10, \\ & \hphantom{\mbox{s.t.}\qquad}9x_{1}+7x_{2}+3x_{3} \geq 10, \\ & \hphantom{\mbox{s.t.}\qquad}x_{1},x_{2},x_{3} \geq 0. \end{aligned}$$

### Example 3

(see [4, 17])

$$\begin{aligned} &\min\quad {\frac{37x_{1}+73x_{2}+13}{13x_{1}+13x_{2}+13}+ \frac{63x_{1}-18x_{2}+39}{13x _{1}+26x_{2}+13}} \\ &\mbox{s.t.}\qquad 5x_{1}-3x_{2} = 3, \\ & \hphantom{\mbox{s.t.}\qquad }1.5\leq x_{1} \leq 3. \end{aligned}$$

### Example 4

(see [16])

$$\begin{aligned} &\min \quad -\biggl(\frac{4x_{1}+3x_{2}+3x_{3}+50}{3x_{2}+3x_{3}+50}+\frac{3x_{1}+4x_{3}+50}{4x_{1}+4x_{2}+5x_{3}+50} \\ &\qquad \qquad {}+\frac{x_{1}+2x_{2}+5x_{3}+50}{x_{1}+5x _{2}+5x_{3}+50}+ \frac{x_{1}+2x_{2}+4x_{3}+50}{5x_{2}+4x_{3}+50}\biggr) \\ &\mbox{s.t.}\qquad 2x_{1}+x_{2}+5x_{3} \leq 10, \\ &\hphantom{\mbox{s.t.}\qquad } x_{1}+6x_{2}+3x_{3} \leq 10, \\ & \hphantom{\mbox{s.t.}\qquad }5x_{1}+9x_{2}+2x_{3} \leq 10, \\ & \hphantom{\mbox{s.t.}\qquad }9x_{1}+7x_{2}+3x_{3} \leq 10, \\ & \hphantom{\mbox{s.t.}\qquad }x_{1},x_{2},x_{3} \geq 0. \end{aligned}$$

### Example 5

(see [16])

$$\begin{aligned} &\min \quad {-\biggl(\frac{3x_{1}+5x_{2}+3x_{3}+50}{3x_{1}+4x_{2}+5x_{3}+50}+ \frac{3x _{1}+4x_{2}+50}{4x_{1}+3x_{2}+2x_{3}+50}+\frac{4x_{1}+2x_{2}+4x_{3}+50}{5x _{1}+4x_{2}+3x_{3}+50} \biggr)} \\ &\mbox{s.t.} \qquad 6x_{1}+3x_{2}+3x_{3} \leq 10, \\ &\hphantom{\mbox{s.t.}\qquad } 10x_{1}+3x_{2}+8x_{3} \leq 10, \\ &\hphantom{\mbox{s.t.}\qquad } x_{1},x_{2},x_{3} \geq 0. \end{aligned}$$

### Example 6

(see [16])

$$\begin{aligned} &\min \quad {\frac{x_{1}+3x_{2}+2}{4x_{1}+x_{2}+3}+ \frac{4x_{1}+3x_{2}+1}{x_{1}+x_{2}+4}} \\ &\mbox{s.t.}\qquad {-}(x_{1}+x_{2}) \leq -1, \\ &\hphantom{\mbox{s.t.}\qquad } x_{1},x_{2} \geq 0. \end{aligned}$$

From Table 1, we can obtain that solving all of the examples by the proposed algorithm yields the $(\varepsilon ,\eta)$-optimal solutions with much better objective function values and being feasible. In addition, for Example 2, it is observed that the computational solution $x^{*}=(0,3.3333,0)$ of Ref. [4] does not satisfy the constraint $x_{1}+6x_{2}+2x_{3} \leq 10$, i.e., $x^{*}$ is infeasible.

Next, we are particularly interested in instances of problem (FP) with a small number of variables and a lager number of ratios. The reason is that this case has many applications, especially in layered manufacturing and material layout [20–22]. In the following, we will show computational results of experimenting on the proposed algorithm for randomly generated problems, which are of the following form:

$$ \mbox{(P)}: \textstyle\begin{cases} \min &\sum_{i=1}^{N}\frac{c_{i}^{\top }y+c}{d_{i}^{\top }y+c} \\ \mbox{s.t.} & Ay\leq b, y\geq 0, \end{cases} $$

where the elements of the matrix $A\in R^{m\times n}$, $c_{i}$, $d_{i}\in R^{n}$ ($i=1,\ldots ,N$) are randomly generated in the unit interval $[0,1]$ and $c\in R$. As for the subdivision operation, except for the proposed operation, we also test the usual bisection operation in branch and bound methods (e.g., [4, 5, 16–18, 23]) for comparison, where their respective computer programs were referred to as adaptive division and bisection. During running procedures, *η* is fixed on 10^−5^, then we obtain a few data up and down; ultimately we choose the average result for running 10 times.

The comparison results between the adaptive division and bisection are shown in Figs. 1–4. Figure 1 illustrates the variedness of average computational time (in seconds) acquired by each item with *c* changing from 10 to 100 in 10 increments when $n=3$, $N=50$, and $\varepsilon =0.05$, by which one can see that the influence of the value of *c* on computational time is slight, hence we let *c* be constant 3 in other experiments. Figure 2 gives the alteration of the average computational time (in seconds) gained by each term with *N* ranging from 50 to 400 when $(n,\varepsilon)=(3,0.05)$, by which one can observe that when $N>150$ the running time increases rapidly, so the effect of variedness of the number of ratios on computational time is biggish. Figure 3 demonstrates the change of the average computational time acquired by each item with *ε* ranging from 0.01 to 0.07 in 0.01 increments. It is obvious that this program is sensitive to changes in *ε*. From numerical results, we can see that the adaptive division acquires less time required by bisection for each program.

For the adaptive division and bisection the computational time of solving examples with larger *N* ranging from 600 to 1500 are listed in Table 2. We can see that even if it takes about seventeen minutes to solve problem (P) of size $(n,N)=(3,1500)$, the adaptive division takes less time required by bisection for each *N*, which confirms the feasibility and availability of the proposed algorithm. We can draw a conclusion that the algorithm has more than enough performance, at least for three dimensions.

How does the algorithm behave for instances with $n>3$? Unfortunately, the performance of the adaptive division rapidly deteriorates with increasing *n*, as shown in Fig. 3. For the same set of instances as in Fig. 1, Fig. 4 shows the variation of average computational time obtained by each program with *n* varying from 10 to 100 when $(N,\varepsilon )$ is fixed at (50, 0.05), from which one can know that even though the running time is large, but the impact of variedness of the number of variables is mild with increasing running time in a nearly linear mode with increasing *N*.

Based on the above results, it is easy to see that the adaptive division has many advantages over the usual bisection in the computation. Additionally, when *n* is not larger than 3, the algorithm can rapidly solve problems, even if *N* takes on values as high as 1500. On the other hand, when the number of variables is as high as 100, with increasing *n*, we need to solve more equations in reduction operation, therefore, it is reasonable that the computational time is larger. Moreover, the lower bound is acquired only by simple arithmetic computations, that is, by executing simple algebraic steps, which is different from the ones used in solving convex or linear programs in common branch and bound methods. Finally, the computation for obtaining ${\hat{\alpha }}_{k}^{i}$ and $\hat{\beta }_{k}^{i}$ is easy by solving the equations with univariate and monotonicity in reduction operation, which is also the main computational cost of the algorithm.

## Results and discussion

In this article, a new division and reduction algorithm is proposed for globally solving problem (FP). First, the original problem (FP) is converted into an equivalent optimization problem (P), in which the objective function is a single variable and the constraint functions are the difference of two increasing functions. Second, several basic operations (i.e., division, deleting, and reduction) are presented for designing a more efficient algorithm to problem (P). Finally, the numerical computational results show the feasibility and efficiency of the proposed basic operations, compared with the usual branch and bound (BB) methods (e.g., [4, 5, 9, 10, 16, 17]). Additionally, as further work, we think the ideas in this article can be extended to the sum of nonlinear ratios optimization problems; for example, the numerator and denominator of each ratio in the objective function to problem (FP) are replaced with a generalized polynomial function, respectively.
